# Mental Nurses in Military Hospitals
*Previous articles appeared on August 26 and September 16.


**Published:** 1916-09-30

**Authors:** 


### THE MATRONS' AND SISTERS' DEPARTMENT.

### MENTAL NURSES IN MILITARY HOSPITALS.*

THE very temperate communication from a correspondent, which appears on p. 564 of THE HOSPITAL for September 16, needs little comment. Our correspondent concedes all that we asserted. The holder of the certificate of the Medico-Psychological Association is very competent to nurse patients who are merely infirm, and the assiduity and skill by which bed-sores are prevented from occurring, in cases peculiarly liable, for months and years together, to these ugly accidents, deserve the recognition that is awarded by the Commissioners of the Board of Control; but such nursing is not surgical nursing, and our correspondent agrees with us that each kind of nurse must stick to her own last. What is meant by preventive nursing, except as it applies to the prevention of bed-sores, we are not quite sure; neither is it clear how a nurse can ward off asthenia, perversion, or syphilis. Our correspondent would have been on firmer ground if he had claimed, as he could justly have claimed, that most mental nurses receive sound training in the prevention of the spread of tuberculosis.

The final question of our correspondent admits of a satisfactory answer. Why, he asks, does one young woman take to hospital nursing while another seeks the asylum? Is it a matter of pecuniary advantage, or one of liberty and discipline, or one of liking for the particular work, or one of moral and possibly physical courage, or, finally, one of intention to offer the most self-denying service to fellow-creatures? It is to be hoped that the motive last suggested is seldom operative, for it is not a wholesome motive, nor does it argue a well-constituted or well-regulated mind. It means in nearly every case that the girl has been disappointed in love, and takes up nursing not from deliberate, well-considered choice, but in a fit of desperation, on impulse, without regard to her suitability for it or her staying power. In many cases the choice of asylum life is made by a girl on the example of relatives or friends who have taken it up before her; in many cases because the asylum is, in a remote country district, the chief employer of female labour, as the factory is in industrial towns. Apart from these motives, the chief influence is, no doubt, the immediate need of earning money. The pay of a probationer in her first year in an asylum is at least double that of a hospital probationer, and in the asylum the uniform is provided by the institution, while the hospital probationer usually has to provide her own. Asylum service offers, therefore, much more immediate advantage than hospital service, and the remote advantages are not less. Other things being equal, therefore, asylum service should be the more attractive, and if it does not, as it certainly does not, attract as good a social class, it is partly from the horror with which madness is still looked upon by those who know nothing about it; partly from the remoteness of asylums, which prevents their claims from being as generally known as those of hospitals; and partly from the reluctance of women of superior social standing to take rank with those who are in this respect lower in the scale. The work in asylums is much less arduous than the work in hospitals; the discipline is less severe; the breakdowns in health among the probationers and nurses are much fewer in proportion to the staff engaged; and recreations and amusements unknown in hospitals form a regular element in the asylum routine. On the other hand, the life in asylums is much more monotonous than the life in hospitals; asylums are usually isolated in country districts, far from the bustle and interests of towns, and it is almost impossible for a nurse in an asylum to keep up the social side of her life, to maintain her friendships, or to keep in touch with her relatives. Still, the asylum has this very great advantage over the hospital, that, after a certain number of years' service in an asylum, a nurse can claim as of right a retiring pension on a fixed scale, an advantage given by some hospitals, but not by all, an advantage which in those hospitals that do give it cannot be claimed of right, and is not on any fixed scale, but is entirely at the discretion of the committee and at the mercy of the hospital funds. All things considered, it is perhaps as much owing to the general ignorance of the advantages of asylum life as to anything else that posts in asylums are not more sought after by a superior class of nurses.

